# Dendritic Cell Control of Immune Responses

**DOI:** 10.3389/fimmu.2015.00042

**Published:** 2015-02-05

**Authors:** Penelope A. Morel, Lisa H. Butterfield

**Affiliations:** ^1^University of Pittsburgh, Pittsburgh, PA, USA

**Keywords:** dendritic cells, immuno response, autoimmune diseases, inflammation, cancer vaccines

Dendritic cells (DC) play a critical role in the initiation of the immune response, acting as sentinels in the tissues, reacting to invading pathogens and then inducing the activation and differentiation of naïve T cells. Since their discovery by Ralph Steinman in the 1970s, we have learned a great deal about DC biology and function. Distinct DC subsets exist in specific tissue niches and within the secondary lymph nodes, and the study of the phenotype and function of DC subsets in mice and humans has been an area of great interest. DC influence the immune response by directing the differentiation of T cells into functional subtypes important for elimination of the relevant pathogen. DC also contribute to disease pathogenesis, such as autoimmunity and cancer, through failures in self-tolerance and promotion of an immunosuppressive environment, respectively. In addition, because of their role in molding T cell responses, DC have been tested in therapeutic settings in both autoimmunity and cancer. In this research topic, 11 articles cover many aspects of current DC biology, ranging from the classification and function of DC subsets, roles of DC in disease pathogenesis to current use of DC in the therapeutic setting.

The topic begins with two reviews of normal DC function; one is focused on the important topic of cross-presentation (1) and the other on human plasmacytoid (p)DC (2). Cross-presentation is the means by which DC are uniquely able to take up, process, and present exogenous antigens in MHC class I molecules and this review (1) describes novel findings on the role of intracellular vesicular traffic in this process and how it is influenced by inflammatory signals. pDCs were first identified as type 1 interferon-producing cells following acute viral infection and have also been shown to have tolerogenic properties. The review by Mathan et al. (2) focuses on interactions between human pDCs and other cells of the immune system with a particular emphasis on cell surface proteins that facilitate these interactions.

The next four articles in this research topic are original research focusing on various aspects of DC function *in vivo* and *in vitro* (3–6). DC maturation is induced by interaction with pathogen-derived molecules such as LPS, but infections also induce production of a large number of cytokines. Work by Hartmann et al. (3) examined the effects of virally induced cytokines on the maturation and function of human DC. These studies identified a set of five cytokines that are critical for the induction DC maturation. In addition, this study emphasizes a systems approach to studying the complex effects of cytokine-induced DC maturation (3). DC play an important role in maintaining tolerance to self-antigens and the second article in this section describes the role of early IL-10 production in induction of tolerance to a self-antigen (6). This article builds on the intriguing observation by the same group that immunization with foreign antigen induces a rapid upregulation of pancreatic enzymes in splenic DC that is correlated with the induction of immunity. The present article shows that immunization with a self-antigen fails to induce pancreatic enzymes and demonstrates a role for IL-10 in this phenomenon (6). Work by Kokulus et al. (5) demonstrates the influence of mild chronic cold stress on the phenotype and function of DC in normal and tumor-bearing mice. These studies highlight the importance of regulating ambient temperature when conducting experiments with experimental animals and the impact of non-physiologic temperature. Chronic human inflammatory diseases are often characterized by changes in circulating monocyte and DC populations. The final article in this section describes a novel flow cytometry panel, using CD4 as a lineage marker that allows the enumeration of monocyte, DC, and lymphocyte populations in a single panel (4). This panel was validated in patients with immunodeficiency, cancer, and inflammatory conditions.

The final group of five reviews highlights the role of DC in either causing or ameliorating disease (7–11). Autoimmune diseases are characterized by a breakdown of self-tolerance followed by an immune response that causes damage to normal tissues. DC play roles at all stages of the autoimmune response and these are outlined in a review that focuses specifically on type 1 diabetes (T1D) (11). This review also highlights the potential for using DC to prevent or treat T1D and discusses the latest clinical trials using or targeting DC in this disease (11). The second review focuses on the many types of immunoregulatory DC and their role in preventing inflammatory conditions such as autoimmunity, transplant rejection, and atopic diseases (9). The last three reviews in this research topic focus on the therapeutic use of DC as cancer vaccines. In the first of these reviews, the importance of tertiary lymphoid structures in the development of effective anti-tumor immunity is discussed (8). The authors show that injection of DC, expressing the transcription factor Tbet, into the tumor stimulates the generation of tertiary lymphoid structures and contributes to tumor eradication. The second provides an overview of the preparation, functional characteristics, and use of human DC in cancer vaccines, with particular emphasis on the culture methods, maturation cocktails, antigen formulation, and routes of delivery that are currently in use (7). The final review in this research topic focuses on the barriers to the generation of effective DC vaccines in cancer patients (10). In particular, this review outlines the challenges posed by immunosuppressive monocyte populations, prevalent in cancer patients, which impede the generation of immunostimulatory DC vaccines.

Thus, this research topic provides a timely overview of some of the recent advances in DC biology and we look forward to many new developments in this exciting field.

## Conflict of Interest Statement

The authors declare that the research was conducted in the absence of any commercial or financial relationships that could be construed as a potential conflict of interest.
